# A Focused Review on Primary Graft Dysfunction after Clinical Lung Transplantation: A Multilevel Syndrome

**DOI:** 10.3390/cells11040745

**Published:** 2022-02-21

**Authors:** Jan Van Slambrouck, Dirk Van Raemdonck, Robin Vos, Cedric Vanluyten, Arno Vanstapel, Elena Prisciandaro, Lynn Willems, Michaela Orlitová, Janne Kaes, Xin Jin, Yanina Jansen, Geert M. Verleden, Arne P. Neyrinck, Bart M. Vanaudenaerde, Laurens J. Ceulemans

**Affiliations:** 1 Laboratory of Respiratory Diseases and Thoracic Surgery (BREATHE), Lung Transplant Unit, Department of Chronic Diseases and Metabolism, KU Leuven, 3000 Leuven, Belgium; jan.vanslambrouck@kuleuven.be (J.V.S.); dirk.vanraemdonck@uzleuven.be (D.V.R.); robin.vos@uzleuven.be (R.V.); cedric.vanluyten@student.kuleuven.be (C.V.); arno.vanstapel@uzleuven.be (A.V.); elena.prisciandaro@uzleuven.be (E.P.); janne.kaes@kuleuven.be (J.K.); xin.jin@student.kuleuven.be (X.J.); yanina.jansen@gmail.com (Y.J.); geert.verleden@uzleuven.be (G.M.V.); bart.vanaudenaerde@kuleuven.be (B.M.V.); 2 Department of Thoracic Surgery, University Hospitals Leuven, 3000 Leuven, Belgium; 3 Department of Respiratory Diseases, University Hospitals Leuven, 3000 Leuven, Belgium; 4 Department of Pathology, University Hospitals Leuven, 3000 Leuven, Belgium; 5 Laboratory of Respiratory Diseases and Thoracic Surgery (BREATHE), Pulmonary Circulation Unit, Department of Chronic Diseases and Metabolism, KU Leuven, 3000 Leuven, Belgium; lynn.willems@kuleuven.be; 6 Department of Cardiovascular Sciences, KU Leuven, 3000 Leuven, Belgium; michaela.orlitova@kuleuven.be (M.O.); arne.neyrinck@uzleuven.be (A.P.N.); 7 Department of Anesthesiology, University Hospitals Leuven, 3000 Leuven, Belgium

**Keywords:** acute lung injury, histology, ischemia-reperfusion injury, lung transplantation, pathophysiology, primary graft dysfunction, radiology, review

## Abstract

Primary graft dysfunction (PGD) is the clinical syndrome of acute lung injury after lung transplantation (LTx). However, PGD is an umbrella term that encompasses the ongoing pathophysiological and -biological mechanisms occurring in the lung grafts. Therefore, we aim to provide a focused review on the clinical, physiological, radiological, histological and cellular level of PGD. PGD is graded based on hypoxemia and chest X-ray (CXR) infiltrates. High-grade PGD is associated with inferior outcome after LTx. Lung edema is the main characteristic of PGD and alters pulmonary compliance, gas exchange and circulation. A conventional CXR provides a rough estimate of lung edema, while a chest computed tomography (CT) results in a more in-depth analysis. Macroscopically, interstitial and alveolar edema can be distinguished below the visceral lung surface. On the histological level, PGD correlates to a pattern of diffuse alveolar damage (DAD). At the cellular level, ischemia-reperfusion injury (IRI) is the main trigger for the disruption of the endothelial-epithelial alveolar barrier and inflammatory cascade. The multilevel approach integrating all PGD-related aspects results in a better understanding of acute lung failure after LTx, providing novel insights for future therapies.

## 1. Introduction

Primary graft dysfunction (PGD) is the clinical syndrome of acute lung injury in the first 72 h after lung transplantation (LTx) defined by hypoxemia and alveolar infiltrates on chest X-ray (CXR) [1]. The pathophysiological hallmarks of PGD are lung edema with decreased compliance, impaired gas exchange and increased pulmonary vascular resistance (PVR) [1,2,3]. These are the result of multiple injuries to the lung grafts, starting in the organ donor and lasting during and after implantation. Improving our understanding of the mechanisms that cause PGD is an important step towards improved outcome after LTx. 

PGD grading, donor and recipient related clinical risk factors and outcome have been reviewed extensively [1,3,4,5,6]. Cellular pathways with current and future therapeutic options have been discussed in detail [7,8,9,10,11,12]. On the other hand, physiological, radiological and histological aspects that define PGD have not received full attention. On many levels, PGD shares its features with acute respiratory distress syndrome (ARDS) [13,14]. PGD can be considered as a distinct phenotype of ARDS with LTx being the clinical insult [15].

The diversity in terminology used to describe the acute lung injury after LTx reflects the many levels that define PGD. Terms as ischemia-reperfusion injury (IRI), reperfusion edema, pulmonary reimplantation response, early allograft dysfunction and primary graft failure have all been used interchangeably to describe acute lung injury after LTx [1,4,10,16,17]. This use of terminology lacks uniformity and clarity.

We aim to provide a focused review on the multilevel aspects of PGD, encompassing the clinical, physiological, radiological, histological and cellular level. If data and literature (both clinical and animal) on features of PGD were missing, complementary research on ARDS filled those gaps.

## 2. PGD at Clinical Level

### 2.1. PGD Grading and Definition

After LTx, clinicians are confronted with patients presenting with hypoxemia and bilateral alveolar infiltrates on CXR. This is called PGD and its heterogeneous presentation required a grading system to estimate the extent of the ongoing acute lung injury.

In an effort to facilitate interpretation of outcome between LTx programs, a first consensus on the definition and grading of PGD was published in 2005 by the International Society for Heart and Lung Transplantation (ISHLT) [2]. The definition was adapted in 2016 [1]. Severity of PGD is graded at four time points after LTx starting from reperfusion of the second lung: T0, T24, T48 and T72. The grading of PGD is determined by the presence of bilateral alveolar infiltrates on CXR and the ratio of partial arterial oxygen pressure (PaO_2_) over the fraction of inspired oxygen (FiO_2_) or P/F ratio (Table 1). The use of postoperative extracorporeal life support (ECLS) should be explicitly reported and taken into account [1].

Clinicians should keep in mind the post-LTx complications that may amplify or confound the grade of PGD. Airway (stenosis or dehiscence of bronchial anastomoses, impaction), vascular (obstruction of the anastomoses), cardiac (left heart failure, dys-synchrony), parenchymal (infection, rejection, aspiration, atelectasis, hemorrhage) and pleural (effusion, hemothorax, pneumothorax, open chest) complications have to be considered [1].

### 2.2. Impact of PGD on Outcome

Epidemiological studies validated the 2005 PGD definition confirming its predictive value for clinical outcome [1]. However, depending on the LTx center, interpretation of the grade and timing of PGD (e.g., PGD grade 3 within 72 h versus PGD grade 3 at 72 h) may result in a different incidence of PGD and related clinical outcome. 

Despite the heterogeneity in clinical and epidemiological research on PGD, it is generally accepted that 25–30% of patients develop PGD grade 3 within 72 h after LTx [18,19,20]. This PGD grade 3 is clearly associated with increased morbidity after LTx and prolongs the duration of mechanical ventilation and hospital stay [21,22]. Thirty- and 90-day mortality is increased in patients with high-grade PGD [18,21,22,23,24]. On the long-term, certain centers reported higher 5- and 10-year mortality in patients who suffered from PGD grade 3 [24,25,26].

The latter could be attributed to the finding that in some centers the incidence of bronchiolitis obliterans syndrome (BOS)—one phenotype of chronic lung allograft dysfunction (CLAD)—was increased in patients who developed PGD grade 3 after LTx [24,25,26,27]. This association could be explained by the strong PGD-related inflammatory cascade that triggers allorecognition and possibly rejection of the lung grafts [28,29,30,31]. The effects of profibrotic mediators like Transforming Growth Factor β have also been suggested to explain the link between PGD and BOS [32]. However, not in all centers this correlation has been observed, including our own.

### 2.3. Clinical Phenotypes of High-Grade PGD

**Figure 1:**
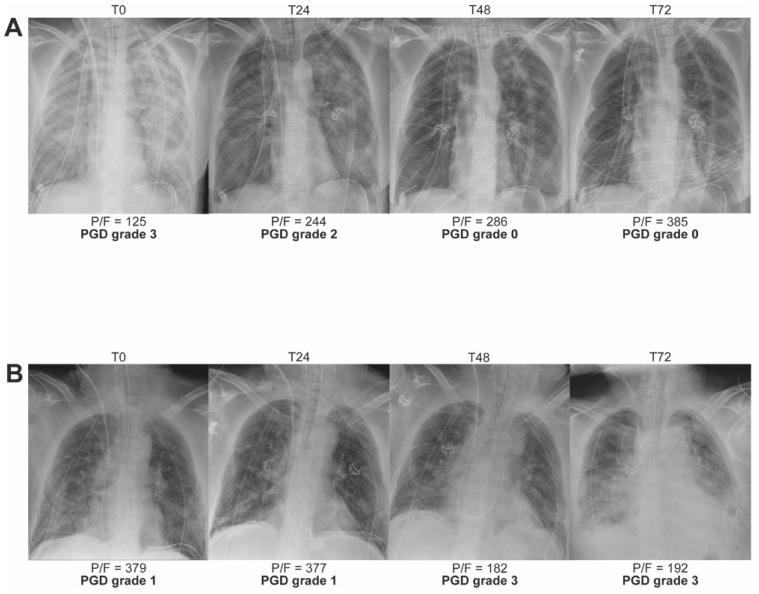
Two phenotypes of PGD grade 3 after LTx with serial chest X-rays (CXR) from two LTx recipients taken at time-point T0/24/48/72 after reperfusion of the second lung. (**A**) CXRs visualizing the phenotype with early transient PGD grade 3. The patient underwent sequential single-LTx via bilateral anterior thoracotomy for end-stage emphysema without extracorporeal life support (ECLS). (**B**) CXRs visualizing the phenotype with increasing and late PGD grade 3. The patient underwent sequential single-LTx via bilateral anterior thoracotomy for end-stage emphysema without ECLS. P/F = PaO_2_ or partial pressure of arterial oxygen/FiO_2_ or fraction of inspired oxygen.

Two phenotypes of PGD can be distinguished, as illustrated in Figure 1 that shows the consecutive CXRs at T0/24/48/72. A first phenotype is characterized by an early and transient PGD grade 3 at T0 that resolves thereafter, resulting in a low-grade PGD at T48 and T72 (Figure 1A). While a second phenotype presents as increasing acute lung injury over time, resulting in a late PGD grade 3 at T48 and T72 (Figure 1B). Remarkably, the second phenotype best predicts early mortality and survival. In a multicenter prospective cohort of 450 lung transplant recipients, PGD grade 3 at T72 better discriminated for 30-day mortality (24.5% for grade 3 at T24 versus 36,4% for grade 3 at T72) [33]. Classification of prospectively collected survival data in 336 patients with PGD grade 3 revealed that recipients suffering from PGD that persisted throughout the first 72 h after LTx had the greatest risk of death (hazard ratio 2.39; 95% CI, 1.57–3.63) [20].

One should consider that the two phenotypes do not correspond to the postoperative course of PGD in all patients. Mixed phenotypes can occur, ranging from consistent PGD grade 0 up to persisting PGD grade 3. These phenotypes reflect what pathophysiological mechanisms should be considered as most prominent in an individual patient. The underlying mechanisms will be further explained in detail hereafter.

### 2.4. Risk Factors, Prevention and Treatment of PGD

Several donor, recipient and procedure-related risk factors have been identified that may impact the likelihood of developing PGD after LTx (Table 2) [3,5]. Therefore, an adequate donor and recipient selection, as well as an optimized preservation and surgical technique are required to prevent the development of PGD after LTx. The emergence of ex-vivo lung perfusion (EVLP) may allow transplant teams to identify unrecognized injury of the grafts before implantation [34,35].

The state-of-the-art evidence for prevention and treatment of PGD has recently been summarized by an ISHLT consensus group statement [34]. No targeted treatments for PGD have been established so far. Similar to ARDS, supportive measures with lung protective ventilation and fluid restriction are the mainstay of treatment. Inhaled nitric oxide and prostaglandins are not routinely recommended but may be useful in the treatment of PGD grade 3. In patients with persistent PGD grade 3 refractory to the aforementioned treatments, and where mechanical ventilation does not achieve adequate gas exchange, postoperative extracorporeal membrane oxygenation (ECMO) is recommended. A more in-depth analysis on treatment and prevention of PGD goes beyond the scope of this focus review and was summarized elsewhere [3,6,34,36,37].

## 3. PGD at Physiological Level

Lung edema—the accumulation of fluid in the extravascular spaces of the lung tissue—is the major determinant of PGD pathophysiology. This edema results in a decreased lung compliance, increased PVR and hypoxemia [1,3,38]. In general, PGD shares these pathophysiological aspects with ARDS and here we describe the common underlying mechanisms [13].

### 3.1. Lung Edema

**Figure 2:**
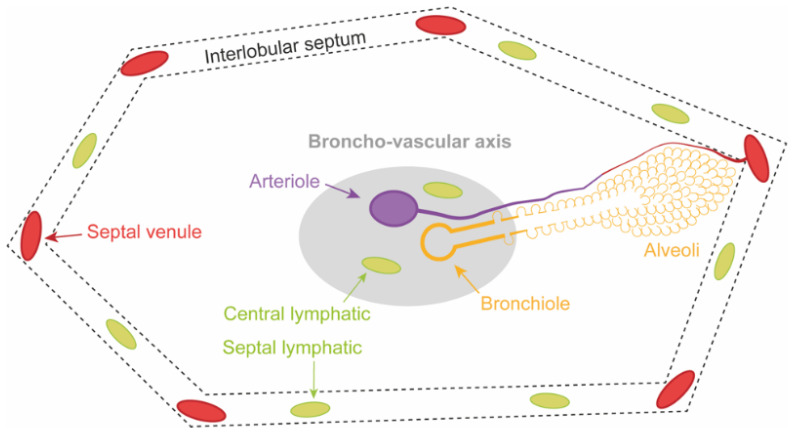
Schematic two-dimensional view on the secondary pulmonary lobule that is polyhedral in shape and 1–2.5 cm in size. A central broncho-vascular axis (bronchiole and arteriole) enters the lobular core and divides towards the alveolar and capillary level. At the periphery, connective tissue structures the interlobular septa that contain the draining venules. The central and septal lymphatic channels run along the broncho-vascular axis and in the interlobular septa, respectively.

Acute lung injury after LTx, predominantly increases the permeability of the alveolo-capillary membrane resulting in lung edema. This membrane forms a barrier between the capillary and alveolar space. The interstitial space is positioned between the endothelial and epithelial lining. Physiologically, fluid that moves across the endothelial lining is drained from the interstitial space by central and septal lymphatics. The secondary pulmonary lobule—shown in Figure 2—is the lung unit that visually explains the structural relation between the broncho-vascular axis, capillaries, alveoli, lymphatics and venules [39]. In addition, the lung has the unique property to actively clear fluid from the alveolar spaces through active sodium transport. This mechanism is referred to as alveolar liquid clearance (ALC). ALC is both protective and predictive for the resolution of lung edema [40].

One should bear in mind that double LTx is performed in a sequential fashion. The recipient native lung on one side is removed, followed by implantation of the first graft. These steps are repeated on the opposite side. If LTx is performed without ECLS, one should anticipate that the firstly implanted graft has to accommodate the entire cardiac output during implantation of the second graft. Normally, the pulmonary vascular bed of the first implanted graft can easily adapt to larger blood volumes. However, when the vascular bed is too small or the adaptation capacity of the vascular bed is reached, pulmonary artery pressures may rise significantly, resulting in right heart dysfunction and ultimately failure. Also hyperperfusion and shear stress induced changes due to acutely increased flow through the post-ischemic lung graft might have an impact on the development of early PGD [41].

During the surgical procedure, test clamping of the pulmonary artery is performed before pneumonectomy. In the occasion that cardiopulmonary instability persists despite optimal management, the implantation of veno-arterial ECMO is required. The role and the indication of routine intraoperative ECMO support for prevention of PGD is a subject of debate. Protocols and indication vary between transplant centers. Therefore, the reported experience cannot be directly compared [42,43]. Until now, data on randomized controlled trials evaluating the effect of intraoperative ECMO support on outcome have not been reported [44].

Furthermore, after graft implantation, lymphatic drainage of the lung grafts is not immediately restored and further impairs fluid homeostasis. Drainage of fluid is therefore largely dependent on ALC and the venous circulation [40,45].

Lung denervation affects the adaptation of pulmonary vasomotor tone to changes in flow, further increasing the capillary hydrostatic pressure gradient over the alveolo-capillary membrane [46].

The clinical phenotype with transient PGD grade 3 at T0 predominantly reflects the short-lived hemodynamic effects of the double LTx procedure (Figure 1A). When the pulmonary artery pressures and capillary hydrostatic pressure after LTx normalize, the edema quickly resolves and PGD severity is downgraded. Based on the underlying mechanisms, transient PGD grade 3 could be defined as the hydrostatic phenotype.

**Figure 3:**
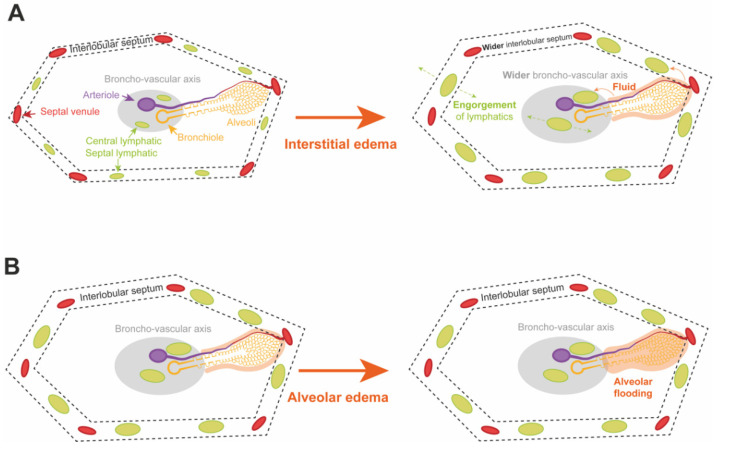
Development of interstitial and alveolar edema in the secondary pulmonary lobule. (**A**) Interstitial edema after LTx is marked by an increase of interstitial fluid that is cleared by the central and septal lymphatic channels that become engorged. The widened aspect of the interlobular septum and broncho-vascular axis explain the reticular pattern observed in chest computed tomography (CT) and clarifies the macroscopic aspect of the interlobular septa that become visibly distended. (**B**) In high-grade PGD after lung transplantation, the stage of interstitial edema progresses to the stage of alveolar edema. The alveolo-capillary membrane permeability and intracapillary hydrostatic pressure continue to increase while alveolar, lymphatic and venous fluid clearance becomes saturated. Flooding of the interstitial and alveolar space explains the ground glass opacities (GGOs) observed on chest CT and represents the edematous and glassy macroscopical aspect of the secondary pulmonary lobules.

Depending on the severity of acute lung injury after LTx, two stages of lung edema can be identified. In a first stage the fluid accumulates in the interstitial space which is drained by an increased lymphatic flow. Figure 3A illustrates this increased flow with engorgement of the central lymphatics along the broncho-vascular axis and the septal lymphatics in the interlobular septum. At this moment, compliance, PVR and gas exchange are not markedly affected [38].

In a second stage, when the ALC and lymphatic flow are saturated, fluid starts to move from the interstitial space to accumulate into the alveolar space. This is called alveolar edema and is illustrated by Figure 3B. At this moment, compliance, PVR and gas exchange are strongly altered and clinically present as high-grade PGD [3,38,40].

### 3.2. Lung Compliance

Compliance—the lung volume change per unit pressure change—decreases as a result of lung edema which is further impaired by absence of surfactant protein [13,38,47].

Interstitial edema decreases compliance by interfering with the elasticity of the lung parenchyma. Small airway collapse—due to the reduced traction from the surrounding parenchyma and the engorgement of the central lymphatics—blocks the aeration of lung parenchyma [38]. 

The shift from interstitial to alveolar edema can be observed in the ventilation parameters as a decline in compliance and tidal volume with increased plateau pressures. Due to the reduction of lung volume, by flooding of the alveolar spaces, compliance further decreases. On top of that, impaired function of surfactant protein—due to increased dilution and reduced production by type II pneumocytes—increases the alveolar wall tension causing alveolar collapse or atelectasis [38].

The most dependent regions (i.e., lowest parts of the lung in relation to gravity) become more consolidated due to alveoli that collapse under the increased lung weight. These consolidated regions strongly reduce the aerated volume of the lung [38,48]. Mechanical ventilation during and after LTx is an additional factor in PGD pathophysiology. The non-dependent lung regions are still ventilated and become overdistended causing volutrauma by mechanical stress on the alveolar walls. On the other hand, regions that are less ventilated are injured by atelectrauma or repetitive opening and closing of the alveoli. These physical forces continue to provoke injury to the alveolo-capillary membrane that was already subject to serious injuries [13,49].

### 3.3. Pulmonary Vascular Resistance

Increased PVR of the lung grafts is encountered during and after the LTx procedure and may cause increased pulmonary vascular pressures. Multiple mechanisms are responsible for the increased PVR. Blood flow to the consolidated lung areas is restricted by hypoxic vasoconstriction. Engorgement of central lymphatics compresses the arterioles in the broncho-vascular axis [38]. In overdistended areas, increased tension on the alveolar wall reduces the capillary flow. In consolidated areas, collapse and flooding of the alveolar spaces and microvascular injury with formation of microthrombi decrease the patency of the capillaries. Furthermore, denervation of the lung grafts affects vasomotor control and may contribute to the increased PVR [13,38,46,50]. High pulmonary artery pressures increase right ventricular afterload and may subsequently lead to intra- and postoperative acute right heart failure [51,52].

### 3.4. Hypoxemia

Through the aforementioned mechanisms resulting in ventilation-perfusion (V/Q) inequality, lung edema culminates in impaired pulmonary gas exchange, which is largely responsible for the development of hypoxemia in PGD. Grading of PGD is based on the severity of hypoxemia by measuring the P/F ratio (Table 1) [1].

The consolidated dependent lung regions are not ventilated but receive up to 50% of total blood flow. In those regions with a very low V/Q ratio, a shunting phenomenon occurs. The non-dependent regions are overventilated and are marked with a high V/Q ratio and high alveolar partial oxygen pressure. However, these regions cannot compensate for the shunting due to the shape of the O_2_-dissociation curve that flattens at higher partial oxygen pressures. Despite the high alveolar partial oxygen pressure, the uptake of oxygen is saturated due to the limited oxygen-binding capacity of hemoglobin. Microthrombi further contribute to V/Q inequality through dead space ventilation [38].

Edema was identified as the driving mechanism behind PGD pathophysiology. Targeting lung edema may restore physiological balance in LTx recipients towards postoperative recovery. 

## 4. PGD at Radiological Level

**Figure 4:**
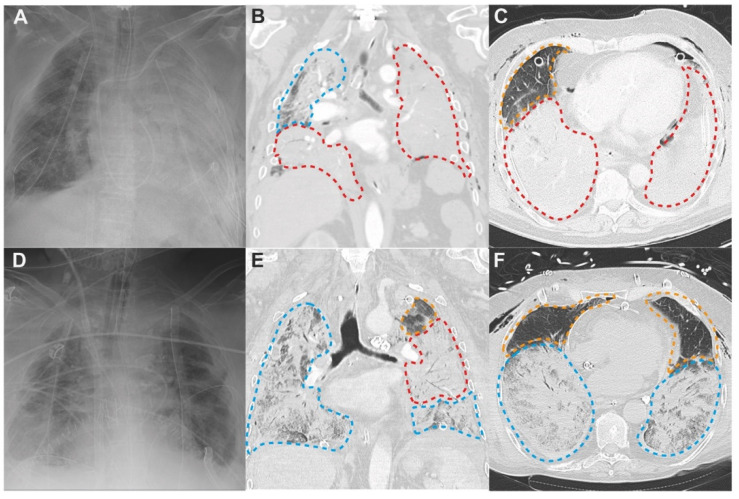
CXR and corresponding chest CT illustrating PGD after LTx. On chest CT three different zones can be discerned: (1) zone defining near normal lung with widened interlobular septa and very limited GGOs (orange dashed lines); (2) zone with marked GGOs, crazy paving and patchy lobular consolidation (blue dashed lines); (3) zone with diffuse consolidation (red dashed lines). (**A**–**C**) CXR and chest CT 0 and 1 h after bilateral lobar LTx (right lung & left lower lobe) via anterior thoracotomy for idiopathic pulmonary fibrosis in a patient with PGD grade 3. High urgent LTx with bridging and intraoperative extracorporeal membrane oxygenation (ECMO) that was continued postoperatively. (**A**) CXR: limited alveolar infiltrates right and complete consolidation of left hemithorax. (**B**) coronal CT: in right lung crazy paving pattern in upper lobe and diffuse consolidation in lower lobe; in left lower lobe diffuse consolidation. (**C**) axial CT: in right lung widened interlobular septa and limited GGOs in upper lobe and diffuse consolidation in lower lobe; diffuse consolidation of left lower lobe. (**D**–**F**) CXR and chest CT 29 and 31 hours after bilateral LTx via clamshell for sarcoidosis in a patient with PGD grade 3. LTx was performed with intraoperative ECMO support that was continued postoperatively. (**D**) CXR: bilateral alveolar infiltrates. (**E**) coronal CT: in right lung mainly crazy paving pattern and patchy lobular consolidations, in left lung GGOs and widened interlobular septa in upper lobe, region of diffuse consolidation with air bronchogram and region of crazy paving pattern with patchy lobular consolidations in lower lobe. (**F**) axial CT: limited GGOs and widened interlobular septa in upper lobes of both lungs, crazy paving pattern and patchy consolidations in lower lobes of both lungs.

At the end of the LTx procedure, the chest is closed. Hence, the lung grafts cannot be directly observed. Clinicians must rely on daily radiological assessment to determine the extent of edema in the lung grafts. In PGD grade 1–3, bilateral alveolar infiltrates are present on CXR (Table 1) [1], however these infiltrates only give a gross estimate on the extent of edema in the lung grafts (Figure 4A,D).

### 4.1. Patterns of PGD on Chest Computed Tomography

Chest computed tomography (CT) provides a more detailed view on the actual extent of ongoing acute lung injury and edema [53,54]. Furthermore, chest CT can provide early detection of post-LTx complications that may confound high-grade PGD.

LTx recipients who underwent chest CT within the first 72 h after LTx, give insight on how edema develops in the lung grafts. In Figure 4, chest CT performed 1 h and 31 h after LTx are showcased and clearly illustrate the distribution of radiological patterns that can be observed in PGD.

The secondary pulmonary lobule—shown in Figure 2—is the structural lung unit that also allows us to better explain the radiological features of PGD [39]. On CT, the different stages of acute lung injury and lung edema in PGD can be discerned at the level of the secondary pulmonary lobule [39]. Engorgement of the septal and central lymphatics in the stage of interstitial edema widens the interlobular septa and broncho-vascular bundles (Figure 3A), resulting in a reticular CT pattern (Figure 4; orange dashed lines). Partial filling of the interstitial and alveolar space results in the early stage of alveolar edema (Figure 3B), causing lobular ground glass opacities (GGOs; Figure 4; blue dashed lines). GGOs and the reticular pattern can appear separately or when combined, present as a crazy paving pattern (Figure 4; blue dashed lines) [45,48,54]. Consolidations on CT arise when fluid and cellular debris fill the alveoli and/or when the alveolar spaces collapse. A patchy pattern of consolidated lobules (Figure 4; blue dashed lines) can evolve to diffuse consolidation of larger lung areas (Figure 4; red dashed lines). Diffuse consolidations are most frequently seen in the gravitationally dependent lung regions [45,54].

### 4.2. Three Lung Zones in PGD

Acute lung injury in ARDS and PGD disrupts the alveolo-capillary membrane in all lung regions, but CT reveals that GGOs and consolidations are not evenly distributed. Similar to CT imaging in ARDS, three different zones can be defined on chest CT imaging for PGD: (1) near-normal zone in non-dependent lung regions (2) GGOs in middle lung regions, and (3) consolidation in dependent lung regions [48,53]. The ‘wet sponge’ theory explains that the air in the dependent regions is squeezed out of the lung under the gravitational force or excess tissue weight of the non-dependent regions. By consequence, under the increased lung weight, the dependent regions collapse and consolidations arise [48]. 

After LTx, CXR and chest CT allow us to indirectly visualize the development of edema in the lung grafts. Visualizing the extent of edema is critical in the daily follow-up of LTx recipients. Therefore, if logistically feasible, the routine use of chest CT 72 h after LTx could be considered [55].

## 5. PGD at Histological Level

The intraoperative aspect of the transplanted lung grafts—observed by the surgeon—provide a macroscopic view on PGD. Lung biopsies sampled in the first hours and days after LTx serve to define the histological patterns of acute lung injury in PGD.

### 5.1. Macroscopy

**Figure 5:**
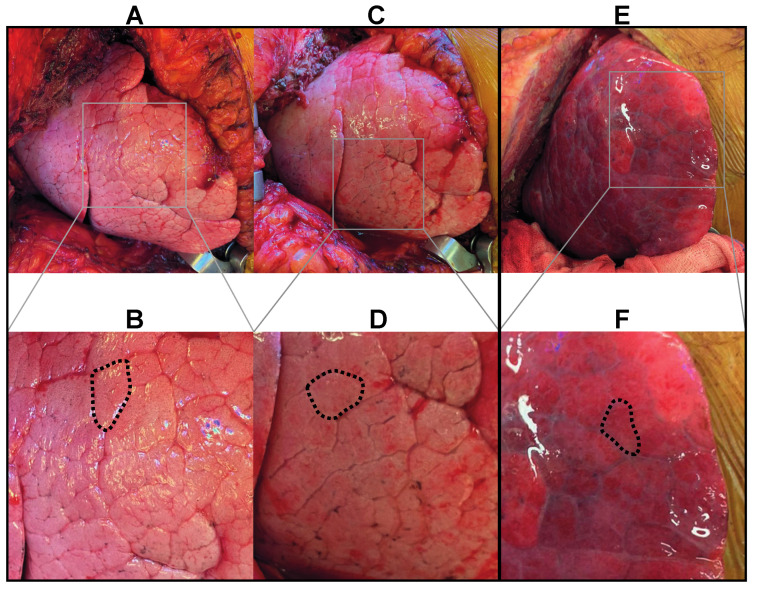
(**A**–**D**) Macroscopic view on the right lung after reperfusion and reinflation in a case without lung edema after LTx (PGD grade 0 T0–T72; PaO_2_/FiO_2_ 91 at T0, 181 at T24, 286 at T48 and 172 at T72) (**A**,**B**) View on the lung immediately after reperfusion and reinflation. The interlobular septa can be appreciated below the visceral pleura and are slender (dashed line). (**C**,**D**) View on the same lung two hours after reperfusion. The septa (dashed line) are clearly distended due to mild interstitial edema but no signs of alveolar edema can be observed. (**E**,**F**) Macroscopic view on the right lung after reperfusion and reinflation in a LTx case with severe intra- and postoperative edema (PGD grade 3 T0–T72; postoperative ECMO at T0–T72). The edematous, glassy and erythematous aspect of the lung with widened interlobular septa (dashed line) can be appreciated.

The secondary pulmonary lobule (Figure 2) allows us to understand the PGD associated macroscopic changes that occur in the first hours after reperfusion [39]. Integrity of the epithelial-endothelial alveolar membrane cannot be visualized directly but the stages of interstitial and alveolar edema can be observed macroscopically. The surgeon can already witness the development of lung edema after reperfusion of the grafts. Figure 5 displays the macroscopic aspect of the lung, immediately after (Figure 5A,B) and two hours after reperfusion (Figure 5C,D). The secondary pulmonary lobules can easily be recognized below the visceral lung surface. In the stage of interstitial edema, lymphatic drainage increases. Widening of the interlobular septa due to engorgement of the septal lymphatics can be macroscopically observed.

In cases of acute lung injury with severe edema and high-grade PGD, development towards the stage of alveolar edema can also be observed intraoperatively. Figure 5E,F showcases the macroscopic aspect of the lung during LTx with severe intraoperative edema. Widening of the interlobular septa was followed by flooding of the alveolar spaces within the secondary pulmonary lobules. Below the visceral surface, the lung turns severely edematous and glassy.

### 5.2. Microscopy

Diffuse alveolar damage (DAD) is the non-specific histological correlate of acute lung injury and is the most frequent manifestation of ARDS [56,57]. It has been suggested that acute lung injury causing PGD after LTx is also characterized by DAD [58,59,60,61]. With transbronchial biopsy, signs of DAD were observed in 30% of a patient cohort within two weeks after LTx [62].

**Figure 6:**
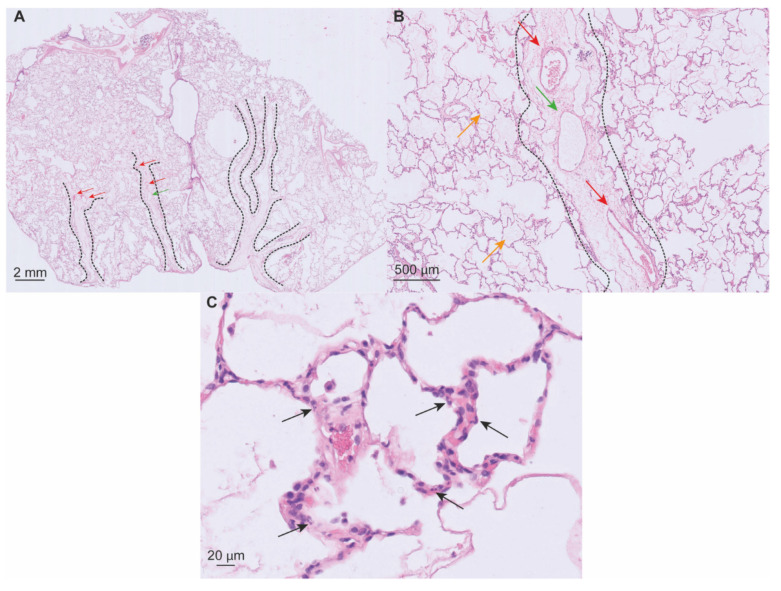
Hematoxylin and eosin stained 5 µm slice of a lung biopsy sampled in a LTx case with severe intraoperative PGD. (**A**) Overview of the section showing widening of interlobular septa containing venules (red arrows) and the lymphatics channels (green arrow) that drain to the subpleural space. (**B**) Alveoli flooded with proteinaceous exudate (orange arrows); widening of interlobular septum (dashed line) with engorgement of lymphatic vessels (green arrow); septal venule (red arrows) (**C**) Mild interstitial inflammation with presence of neutrophils (black arrows).

Data on histological findings early after LTx are scarce. Considering PGD as a distinct phenotype of ARDS, histological findings of DAD in the first days after LTx are similar. In Figure 6, sections from an intraoperative biopsy sampled in a case of early high-grade PGD are displayed. The microscopical sections in this case show us the earliest histological features of DAD in LTx.

The acute or exudative stage of DAD typically occurs during the first week after injury. The organizing stage starts to appear after the first week. Features of these stages can also be found together. In the first two days, interstitial (Figure 6A) and alveolar edema (Figure 6B) progressively develop. Leukocytes appear in the alveolar walls, but interstitial inflammation is not pronounced in DAD (Figure 6C). Four to five days after the onset of DAD, hyaline membranes—eosinophilic structures composed of plasma proteins and cellular debris stuck to the alveolar walls—are most prominent. One week after the lung injury, the organizing stage of DAD appears as a result of tissue repair. In the alveolar walls, fibroblasts proliferate and deposit an extracellular matrix. Areas of alveolar collapse and atelectasis are observed and are a result of impaired surfactant protein function. Hyperplastic pneumocytes repopulate the denuded alveolar membrane. Microthrombi may be present due to coagulopathy localized in the capillaries and are seen during the acute and organizing stage [13,14,50,56].

This histological description of PGD on the macro- and microscopical level serves as an interface that connects the earlier defined physiological and radiological features; and the associated cellular pathways that will be described in the next section.

## 6. PGD at Cellular Level

At the cellular level, ischemia-reperfusion injury (IRI) is considered the major determinant of PGD. Although, IRI and PGD are interchangeably used as synonyms, IRI should be considered as one of the underlying mechanisms responsible for the development of lung edema in the clinical syndrome of PGD.

Also at the cellular level, PGD shares features with ARDS. Lung IRI after LTx is the insult responsible for microvascular injury, disruption of the endothelial-epithelial alveolar membrane, alveolar fluid clearance, and surfactant production that are found in ARDS [13,14].

The sudden interruption of flow in the lung (*ischemia*), cold storage of the graft and the implantation followed by sudden *reperfusion* of the organ trigger a cascade of events that converge to acute *injury* of the lung. This injury strongly increases the permeability of the endothelial-epithelial alveolar membrane that separates the alveolar space and capillary lumen [7,9,10,63].

**Figure 7:**
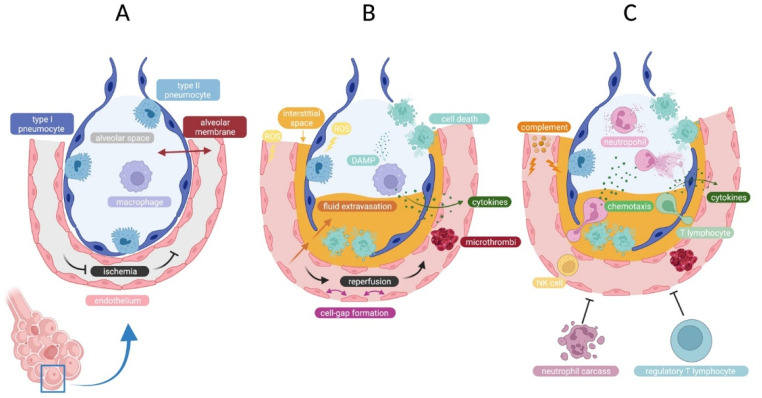
Overview of ischemia-reperfusion injury (IRI) at the cellular level in the lung graft during and after LTx. (**A**) Cessation of flow or ischemia in the alveolar capillaries sets off IRI. (**B**) Phase of donor-derived inflammation initiates the innate immune response after reperfusion. (**C**) Phase of recipient-derived inflammation that further precipitates injury in the lung. (Created with BioRender.com). DAMP = damage associated molecular pattern; NK = natural killer; ROS = reactive oxygen species.

IRI in LTx is the result of a complex interplay between a multitude of cell types connected by innumerous pathways [8,9,10,12,63]. Our understanding of lung IRI is based on a combination of clinical and animal research [11]. IRI can be defined as three interacting events which are represented in Figure 7: (1) vascular endothelial dysfunction (2) alveolar epithelial injury, and (3) inflammation. These events are set off by ischemia and continue to define IRI during and after reperfusion [64,65].

Endothelial dysfunction and inflammation in the capillaries cause microvascular injury. Cell-gap formation by cytoskeleton rearrangement [66] and expression of transmembrane ion channels [67] increase the capillary permeability. Dysregulated coagulation causes platelet aggregation and formation of microthrombi with release of vasoactive substances that further propagate endothelial injury [63,68,69]. The glycocalyx is a mesh-like layer composed of proteoglycans that are anchored to the endothelial cells, and project in the capillary lumen. Damage to the glycocalyx is observed in IRI and contributes to endothelial dysfunction and barrier disruption [70,71,72,73,74].

Ischemia-reperfusion triggers the production of reactive oxygen species (ROS) [75,76]. Ischemia with interruption of flow and reperfusion trigger mechano-signaling pathways that promote enzymatic production of ROS in the endothelium [77]. ROS play a critical role in lung IRI by increasing endothelial cell permeability [67], promoting the release of pro-inflammatory cytokines, causing oxidative stress with direct cell injury, and driving expression of cell adhesion molecules on the luminal cell surface of the endothelium [78].

Widespread damage and inflammation in the alveoli result in epithelial cell death [79,80]. Injury and cell death of type I pneumocytes disrupts alveolar fluid homeostasis and causes basement membrane denudation. Decreased production of surfactant due to loss of type II pneumocytes leads to alveolar collapse [13].

From the moment of ischemia (Figure 7A), the disturbance of metabolic supply and demand distorts epithelial and endothelial metabolism, leading to cellular and mitochondrial Na^+^, K^+^ and Ca^2+^ ion imbalance [81,82]. Mitochondrial swelling, oxidative stress and pro-inflammatory signaling eventually lead to controlled and uncontrolled cell death [83,84,85,86]. Necroinflammation—inflammation associated with cell death—follows and is the result of endogenous ligands or damage associated molecular patterns (DAMPs) that are released from the dying cells [87,88,89].

The events at the level of the endothelium and epithelium are strongly intertwined with inflammation. Endothelial dysfunction and epithelial injury set off the immune response that further injures the endothelial-epithelial alveolar membrane.

DAMPs bind to pattern recognition receptors (PRR) [89,90,91,92,93] and trigger donor antigen-presenting cells (APC) including dendritic cells [94], macrophages [95,96] and monocytes [97]. PRR-DAMP binding leads to a pro-inflammatory cellular state in which activated transcriptional factors [87,89] upregulate expression of cytokines (IL-1β, IL-2, IL-6, IL-17, TNF-α) [89,98,99] and chemokines (IL-8, CXCL-2) [97,100].

ROS production, necroinflammation and DAMP-PRR binding form the first wave of donor-driven inflammation that engulfs the lung graft with pro-inflammatory mediators that lead to chemotaxis of recipient neutrophils (Figure 7B) [100,101,102,103,104]. A second wave of inflammation is characterized by the recipient’s innate-immune activation (Figure 7C).

Chemotactically attracted neutrophils easily bind to the overexpressed cell adhesion molecules/integrins on the endothelial surface [105,106]. At the site of inflammation, neutrophils precipitate injury through release of barrier degrading enzymes, ROS and neutrophil extracellular traps (NETs) [69,107,108,109].

Humoral mediators of the innate immune system also contribute to IRI. Uncontrolled activation of the complement cascade causes increased vascular permeability and cell injury [110,111,112].

Natural killer (NK) cells and T-lymphocytes contribute to innate immune inflammation through antigen-independent cytokine and chemokine production [103,113]. Furthermore, early innate immune signals trigger the lymphocytes that effectuate the later adaptive immune response [24,28,30,114].

Alloantigens are presented by APCs to recipient T- and B-lymphocytes. T-lymphocytes infiltrate the grafts and cause lung injury leading up to cellular acute rejection. Activated B-lymphocytes are responsible for the antibody-mediated rejection. Lymphocyte activation and proliferation are partly counteracted by the administration of high dose immunosuppression after LTx [31].

The innate-adaptive interface possibly links the severity of PGD to the later development of CLAD [29,30]. The clinical phenotype with increasing and late PGD grade 3 reflects the activation of the innate and adaptive immunity that progressively injures the endothelial-epithelial alveolar membrane in the days following LTx. Based on the underlying mechanisms, increasing PGD grade 3 could be defined as the immunological phenotype (Figure 1B). Acute rejection due to strong activation of adaptive immunity can amplify acute lung injury and therefore the grading of the immunological PGD phenotype.

Neutrophils and lymphocytes play important roles in damaging the lung graft after LTx. Conversely, neutrophils mediate tissue repair through their apoptotic carcasses that are cleared by macrophages, a process called efferocytosis [115]. Regulatory T-lymphocytes inhibit innate inflammation [116], increase epithelial repair [117] and promote graft tolerance [118]. Damage pathways of IRI are hereby counteracted by neutrophil- and lymphocyte-mediated tissue homeostasis and repair [40,115,116,117,119]. Balance between these pathways determines the severity of lung IRI, and finally the resolution of PGD after LTx.

## 7. Conclusions

Level-by-level we have reviewed the features of PGD after LTx. PGD can be considered as a distinct phenotype of ARDS elicited by the LTx procedure. The course of PGD grading over time can be classified into a hydrostatic/transient and immunological/increasing phenotype or a mix of both. The pivotal role of edema in PGD renders it a crucial target for future prevention and treatment strategies. Implementation of chest CT would improve our understanding on the spatial distribution of edema. Increasing the sampling of lung biopsies will add to the scarce knowledge on PGD histology. Our understanding of PGD at the cellular level holds the key for future therapies that might prevent disruption of the endothelial-epithelial alveolar membrane and dampen the inflammatory cascade, responsible for inferior outcome after LTx.

We encourage researchers to consider all PGD levels in future studies. This bigger picture will lead to new insights that can guide us towards innovative strategies to tackle the challenge of PGD after LTx.

## Figures and Tables

**Table 1:** Grading of primary graft dysfunction (PGD) after lung transplantation (LTx) according to the 2016 definition of the International Society for Heart and Lung Transplantation (ISHLT).

Grade	Bilateral Alveolar Infiltrates on Chest X-ray	PaO_2_/FiO_2_ Ratio
PGD grade 0	No	Any
PGD grade 1	Yes	>300
PGD grade 2	Yes	200–300
PGD grade 3	Yes	<200

PGD is graded at 4 time-points over the first 72 h after LTx, i.e., every 24 h starting at reperfusion of the second lung (T0, T24, T48 and T72) [1]. FiO_2_ = fraction of inspired oxygen; PaO_2_ = partial pressure of arterial oxygen; PGD = primary graft dysfunction.

**Table 2:** Clinical risk factors for PGD after LTx.

Category	Risk Factors
Donor	Age > 45 years or <21 years
	Female sex
	History of smoking
	Mechanisms of death: aspiration, head trauma
	Hemodynamic instability after brain death
	Prolonged mechanical ventilation
Recipient	BMI > 25
	Female sex
	Diagnosis: idiopathic pulmonary fibrosis, idiopathic pulmonary hypertension, secondary pulmonary hypertension, sarcoidosis
	Elevated pulmonary artery pressure at time of surgery
Procedure	Prolonged ischemic time
	Single lung transplantation
	Use of cardiopulmonary bypass
	Administration > 1 L packed red blood cells
	FiO_2_ > 0.4 at reperfusion

Adapted from references [3,5]. BMI = body mass index.

## Data Availability

Not applicable.
